# Cyclosporin A enhances neural precursor cell survival in mice through a calcineurin-independent pathway

**DOI:** 10.1242/dmm.014480

**Published:** 2014-08

**Authors:** Nadia Sachewsky, Jessica Hunt, Michael J. Cooke, Ashkan Azimi, Taraneh Zarin, Carween Miu, Molly S. Shoichet, Cindi M. Morshead

**Affiliations:** 1 Institute of Medical Science, University of Toronto, Medical Science Building, 1 King’s College Circle, Toronto, ON M5S 1A8, Canada.; 2 Department of Surgery, University of Toronto, Donnelly Centre, 160 College Street, Toronto, ON M5S 3E1, Canada.; 3 Institute of Biomaterials and Biomedical Engineering, University of Toronto, Donnelly Centre, 160 College Street, Toronto, ON M5S 3E1, Canada.

**Keywords:** Cyclosporin A, Adult neural precursors, Mitochondrial permeability transition pore formation, Cyclophilin D, Calcineurin-independent signaling, FK506, Stroke

## Abstract

Cyclosporin A (CsA) has direct effects on neural stem and progenitor cells (together termed neural precursor cells; NPCs) in the adult central nervous system. Administration of CsA *in vitro* or *in vivo* promotes the survival of NPCs and expands the pools of NPCs in mice. Moreover, CsA administration is effective in promoting NPC activation, tissue repair and functional recovery in a mouse model of cortical stroke. The mechanism(s) by which CsA mediates this cell survival effect remains unknown. Herein, we examined both calcineurin-dependent and calcineurin-independent pathways through which CsA might mediate NPC survival. To examine calcineurin-dependent pathways, we utilized FK506 (Tacrolimus), an immunosuppressive molecule that inhibits calcineurin, as well as drugs that inhibit cyclophilin A-mediated activation of calcineurin. To evaluate the calcineurin-independent pathway, we utilized NIM811, a non-immunosuppressive CsA analog that functions independently of calcineurin by blocking mitochondrial permeability transition pore formation. We found that only NIM811 can entirely account for the pro-survival effects of CsA on NPCs. Indeed, blocking signaling pathways downstream of calcineurin activation using nNOS mice did not inhibit CsA-mediated cell survival, which supports the proposal that the effects are calcinuerin-independent. *In vivo* studies revealed that NIM811 administration mimics the pro-survival effects of CsA on NPCs and promotes functional recovery in a model of cortical stroke, identical to the effects seen with CsA administration. We conclude that CsA mediates its effect on NPC survival through calcineurin-independent inhibition of mitochondrial permeability transition pore formation and suggest that this pathway has potential therapeutic benefits for developing NPC-mediated cell replacement strategies.

## INTRODUCTION

Central nervous system (CNS) injury and disease often lead to severe functional deficit, with no regenerative treatment currently available. The discovery of neural stem cells (NSCs) in the adult brain has led to much effort in exploring the potential of these cells in the development of therapies to treat the injured CNS. Clinically relevant molecules that modify the properties of neural stem and progenitor cells (termed neural precursor cells; NPCs) could well provide a foundation for regenerative strategies that aim to replace lost and damaged neural cell types in the injured CNS. Towards this end, we have previously demonstrated that cyclosporin A (CsA) has direct effects on NPCs, specifically enhancing cell survival (Hunt et al., 2010), with no effects on cell cycle kinetics or NPC differentiation. Moreover, CsA administration *in vivo* expands the size of the NPC pool (Hunt et al., 2010) and promotes functional recovery in a model of stroke (Erlandsson et al., 2011). Together, these findings suggest that the intracellular targets of CsA could provide novel therapeutic targets for the development of NPC-mediated regenerative strategies.

**Fig. 1.**
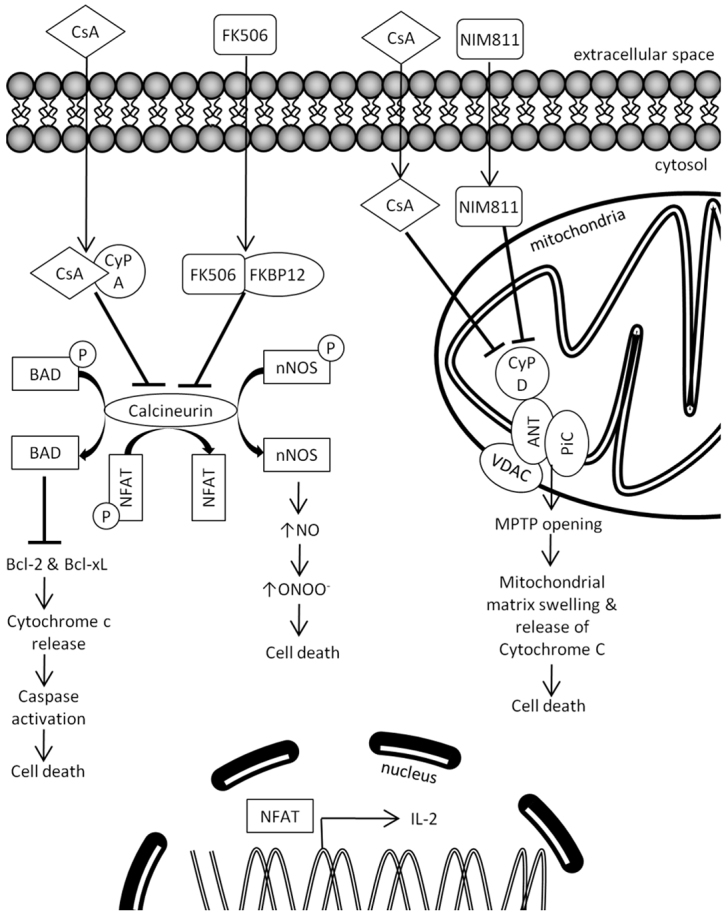
**CsA can influence cell survival through calcineurin-dependent and calcineurin-independent pathways.** CsA and FK506 are calcineurin blockers, but they bind different cytosolic proteins called cyclophilin and FKBP, respectively. Calcineurin inhibition can prevent cell death through a BAD and/or NOS pathway. First, dephosphorylated BAD protein is pro-apoptotic and initiates apoptosis. It binds and blocks anti-apoptotic proteins (Bcl-2 and Bcl-xL) and prevents them from stopping apoptosis. Bcl-2 and Bcl-xL proteins inhibit cytochrome c release through the mitochondrial pore and therefore inhibit the activation of the cytoplasmic caspase cascade caused by cytochrome c release. Alternatively, dephosphorylated nNOS produces nitric oxide. Nitric oxide can interact with superoxide anion to form peroxynitrate, which interacts with either DNA or proteins via oxidative reactions that can trigger oxidative injury and necrosis or apoptosis. CsA can also influence cell survival through a calcineurin-independent pathway. MPTP is a protein pore formed in the inner membrane of the mitochondria that can lead to mitochondrial swelling and cell death through apoptosis or necrosis. The pore is formed by ANT, PiC, VDAC and CyP D. The pore opening raises the permeability of the mitochondrial inner membrane and allows influx of cytosolic molecules into the mitochondrial matrix, increases matrix volume, disrupts membrane potential and ATP synthesis and causes loss of cytochrome C, a component of the electron transport chain. CyP D regulates pore opening and CsA binds and inhibits CyP D. NIM811 blocks CyP D without affecting calcineurin. ANT, adenine nucleotide translocase; BAD, Bcl-2-associated death promotor; Bcl-xL, B-cell lymphoma-extra large; Bcl-2, B-cell lymphoma 2; CyP A, cyclophilin A; CyP D, cyclophilin D; MPTP, mitochondrial permeability transition; VDAC, voltage-dependent anion channel.

Understanding the mechanism by which CsA selectively enhances NPC survival, and avoiding the undesirable systemic immunosuppression, has implications for the development of stem cell-based therapies for neurorepair. CsA is a small, lipophilic, cyclic polypeptide immunosuppressant routinely used to treat autoimmune disorders and prevent graft rejection (Borel et al., 1976; Borel et al., 1977; Faulds et al., 1993). CsA freely crosses the plasma membrane and binds to several receptors from a family of peptidyl-prolyl *cis-trans* isomerases known as cyclophilins (Fischer et al., 1989; Handschumacher et al., 1984; Takahashi et al., 1989). The immunosuppressive effect of CsA on T-lymphocytes is mediated by CsA binding to cyclophilin A (Walsh et al., 1992), creating a drug-receptor complex that binds and inhibits calcineurin, a Ca^2+^/calmodulin-activated phosphatase (Griffith et al., 1995; Kissinger et al., 1995). As shown in Fig. 1, calcineurin inhibition prevents transcription of interleukin 2 (IL-2), a cytokine responsible for T-lymphocyte propagation (Flanagan et al., 1991; Fruman et al., 1992; Kay et al., 1983). Further, blocking calcineurin inhibits dephosphorylation of both neuronal nitric oxide synthase (nNOS) (Kaminska et al., 2004) and the pro-apoptotic protein, Bcl-2 Associated Death promoter (BAD) (Huang et al., 2005), to effectively promote cell survival. Similarly, CsA promotes cell survival via a calcineurin-independent pathway by binding to mitochondrial cyclophilin D (Baines et al., 2005), which blocks mitochondrial permeability transition (MPT) pore formation and inhibits cytochrome c release, a potent apoptotic stimulation factor (Basso et al., 2005). Hence, both calcineurin-dependent and calcineurin-independent pathways can potentially mediate the CsA-mediated survival of NPCs observed *in vitro* and *in vivo*.

Herein, we sought to determine the mechanism by which CsA enhances cell survival in adult-derived NPCs. We used inhibitors of calcineurin such as FK506 and cyclophilin A inhibitors and blocked the downstream effectors of calcineurin using nNOS knockout mice (*nNOS*^−/−^). To examine the calcineurin-independent pathway we used an analog of CsA, NIM811, which binds cyclophilin D and blocks MPT pore formation (Waldmeier et al., 2002). Both *in vivo* and *in vitro* assays revealed that the pro-survival effects of CsA were entirely accounted for by NIM811. Moreover, the administration of NIM811 led to functional recovery in a model of stroke, identical to the effects observed following CsA administration. Interestingly, we found that FK506 modifies NPC survival indirectly by acting on non-NPCs. Hence, the pro-survival effect of CsA on NPCs is calcineurin-independent and the result of inhibition of MPT pore formation.

**TRANSLATIONAL IMPACT**

**Clinical issue**

The development of stem cell-based therapies for the treatment of trauma or disease is an exciting prospect in regenerative medicine. There are two approaches for using stem cells for neurorepair: (1) exogenous therapies involving the transplantation of neural stem and progenitor cells and (2) endogenous therapies that activate resident stem cells and promote self-repair of the injured or diseased CNS. Previous studies have demonstrated that cyclosporin A (CsA), a widely used immunosuppressant, is able to promote the survival of neural precursor cells (NPCs) without modifying their lineage potential or proliferation kinetics; however, the pro-survival effects are not mediated by immunosuppressive molecules such as IL-2. We have previously demonstrated that CsA administration effectively promotes functional recovery following stroke, indicating therapeutic potential. Understanding the mechanism by which CsA selectively enhances NPC survival and avoids undesirable systemic immunosuppression could inform the development of effective stem cell-based therapies for neurological disease.

**Results**

Calcineurin is a Ca^2+^/calmodulin-activated phosphatase with a role in immune cell activation. The immunosuppressive effects of CsA are thought to be mediated through inhibition of calcineurin. Here, the authors sought to determine whether CsA acts in a calcineurin-independent or -dependent fashion to promote NPC survival. Clonally derived colonies of NPCs, known as neurospheres, were generated to provide an *in vitro* system for examining the relative importance of calcineurin-independent and -dependent pathways. Exposure of cultured neurospheres to an immunosuppressive molecule that inhibits calcineurin had no effect on cell survival. Furthermore, a non-immunosuppressive derivative of CsA, N-methyl-4-isoleucine (NIM811), was able to completely mimic the pro-survival effects of CsA on NPCs *in vitro*. The authors provide evidence that the enhancement of survival by NIM811 is mediated by binding to cyclophilin D, which inhibits mitochondrial permeability transition pore formation. Finally, they show that NIM811 infusion promotes functional recovery in a mouse model of stroke.

**Implications and future directions**

Overall, these data indicate that CsA promotes NPC survival via a calcineurin-independent pathway. This work thereby provides mechanistic insight into the action of CsA, specifically its ability to selectively enhance NPC survival without triggering immunosuppression. This finding, together with the demonstration of *in vivo* efficacy in a model of stroke, further highlights the potential of CsA to promote stem cell-based neural repair in a clinical setting. The study demonstrates how repurposing of FDA-approved drugs provides interesting venues by which new therapies can be established.

## RESULTS

### FK506 (Tacrolimus) does not have direct effects on neural precursor cell survival

Neural precursor cells reside in the subependyma of the adult forebrain lining the lateral ventricles where they proliferate to generate rapidly dividing progeny that undergo cell death or migrate to the olfactory bulb where they differentiate into olfactory interneurons (Lois and Alvarez-Buylla, 1994; Morshead et al., 1994; Morshead et al., 1998). *In vitro*, NSCs proliferate to generate clonally derived colonies of NPCs (termed neurospheres) in the presence of growth factors (Morshead et al., 2002; Reynolds and Weiss, 1992). In a first series of experiments, we looked at whether CsA promoted NPC survival via a calcineurin-dependent pathway using FK506, also known as Tacrolimus, which is known to inhibit calcineurin. FK506 is structurally unrelated to CsA; however, it has the same immunosuppressive effects at the molecular and cellular level (Schreiber and Crabtree, 1992). CsA and FK506 inhibit calcineurin following binding with cytosolic binding proteins, cyclophilin A and FKBP-12, respectively (Schreiber, 1991) (Fig. 1). Calcineurin inhibition by either CsA or FK506 might enhance cell survival through two downstream pathways: nNOS and/or expression of the pro-apoptotic molecule BAD. Both nNOS and BAD signaling pathways are activated by the phosphatase activity of calcineurin; hence, blocking calcineurin would effectively enhance cell survival.

**Fig. 2.**
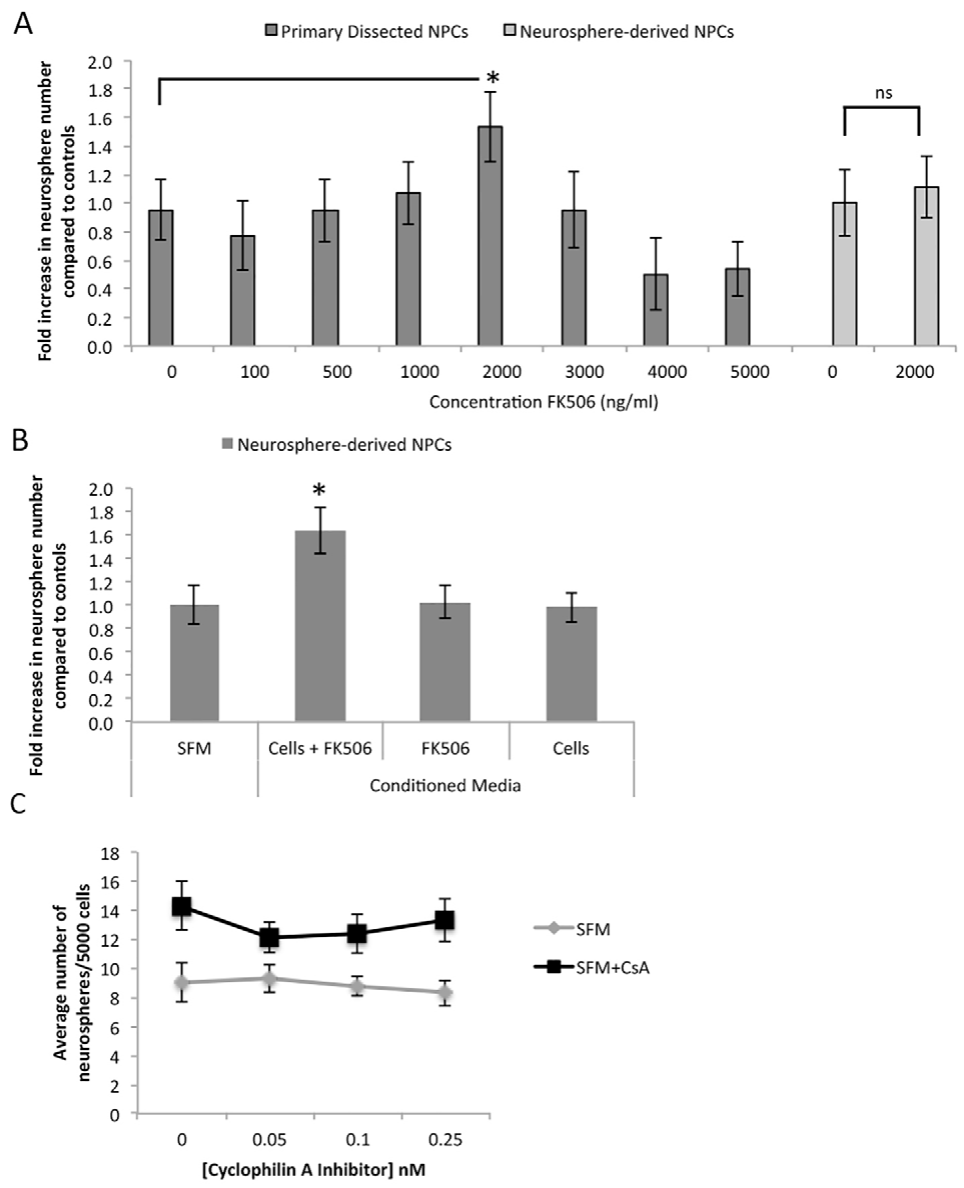
**FK506 increased neurosphere numbers for subependyma-derived cells, but not neurosphere-derived cells.** (A) FK506 was added to subependyma-derived (primary) cell cultures (*n*≥3 independent experiments, 3 mice/experiment) and neurosphere-derived cells (*n*=4 independent experiments). For subependyma-derived cells (dark gray bars), neurosphere numbers increased 1.6-fold at 2000 ng/ml FK506. Neurosphere-derived cells (light gray bars), were not affected by FK506. (B) When neurosphere-derived cells were plated in conditioned medium derived from 24-hour primary subependyma cultures (30 cells/μl) with 2000 ng/ml FK506 (*n*=2 independent experiments, 2–3 animals/experiment), a significant 1.6-fold increase in neurosphere numbers was observed compared with controls (SFM). No increase in neurosphere numbers was seen using 24-hour conditioned medium from cultures of FK506 alone (no cells) (‘FK506’) or 24-hour medium prepared with subependyma-derived cells only (no FK506) (‘Cells’) (*n*=4 independent experiments). (C) Primary subependyma cells were cultured in the presence or absence of CsA with the addition of a cyclophilin A inhibitor at various concentrations (*n*=4 independent experiments, 2–3 mice/group/experiment). A significant increase in neurosphere formation was seen in the presence of CsA (two-way ANOVA, *F=*16.85, *P*=0.0002), which was not inhibited by cyclophilin A inhibitor (two-way ANOVA, *F*=0.64, *P*=0.7); *P<0.05.

We predicted that if CsA enhances NPC survival via calcineurin then FK506 would have similar effects on NPCs, increasing the numbers and size of neurospheres. To test this hypothesis, we established neurosphere cultures from adult brains using standard conditions in the presence or absence of FK506 (0–5000 ng/ml). After 7 days in culture, the number and size of neurospheres from each condition was measured. The total number of neurospheres reflects the number of stem cells, whereas the size of an individual neurosphere reflects the number of progenitors that make up the bulk of the neurosphere (Hunt et al., 2010; Morshead et al., 2002). Although the FK506-FKBP-12 complex has higher binding affinity for calcineurin than CsA (Fruman et al., 1992), it took a 20-fold larger dose to observe a significant increase in neurosphere numbers (1.5-fold increase at 2000 ng/ml FK506 relative to control conditions; 12±3 spheres versus 18±3 spheres for control versus FK506; *P*<0.05) (Fig. 2A). This increase in response to FK506 is significantly less than the maximum observed for CsA, where a 1.7-fold increase was seen (Hunt et al., 2010). Unlike CsA, exposure to FK506 did not increase neurosphere size (average diameter was 90±8 μm versus 101±12 μm for control and 2000 ng/ml FK506, respectively; *P*>0.05). The FK506-mediated increase in numbers of neurospheres (but not size) suggests a differential effect on neural stem versus progenitor cells.

Because primary neurosphere cultures derived from adult brain dissections contain NPCs as well as mature brain-derived cells (i.e. neurons, glia, endothelial cells, microglia), the increased number of neurospheres could be due to an indirect effect of FK506 on these contaminating cells that subsequently modify the behavior of NPCs. We tested this in two ways. First, we established cultures of pure populations of neurosphere-derived NPCs and exposed them to FK506. Neurosphere-derived NPC cultures consist of only stem and progenitor cells, without the contaminating niche cells seen in primary dissections (Hunt et al., 2010; Piccin and Morshead, 2011). Therefore, the use of neurosphere-derived NPCs allowed us to examine the direct effects of FK506 on NPCs without the indirect contribution of niche cells. Control neurospheres (i.e. previously unexposed to FK506) were collected, dissociated into single cells and plated in growth factor-supplemented medium with or without 2000 ng/ml FK506. The numbers and size of neurospheres were assayed 7 days later. Unlike the primary dissected tissue, FK506 treatment did not result in an increase in numbers of neurospheres (25±3 neurospheres versus 27±4 neurospheres, control versus FK506 treatment; *P*>0.05) (Fig. 2A). Because FK506 does not increase neurosphere numbers in pure populations this suggests that FK506 does not have direct effects specifically on NSCs. Hence, the 1.5-fold increase observed in primary cultures might be due to an indirect effect of FK506 on non-NPCs.

To directly test whether the increased number of primary neurospheres in the presence of FK506 is mediated by an indirect effect of FK506 on non-NPCs that subsequently increase NSC survival, we performed experiments using conditioned media (CM) established from primary dissections. We predicted that if contaminating non-NPCs in primary cultures were secreting signaling molecules in response to FK506 that influence NPC behavior, then CM would lead to increased numbers of neurospheres from pure populations of NPCs. Indeed, when neurosphere-derived NPCs were cultured with CM derived from primary dissected subependymal tissue exposed to 2000 ng/ml FK506 for 24 hours, we observed a significant 1.6-fold increase in neurosphere numbers in NPCs (*P*<0.05) (Fig. 2B). No increase in neurospheres was observed in any of the control conditions, including CM derived from 24-hour cultures containing (1) FK506 only (no cells), (2) subependyma-derived cells only and (3) medium only (Fig. 2B). These data indicate that FK506 indirectly affects neurosphere formation by acting on non-NPC cells that subsequently release factors that modify NSC behavior.

To further confirm that the pro-survival effects of CsA were not occurring through a calcineurin-dependent pathway, we used a cyclophilin A inhibitor (CAS 1186372-20-2) to block signaling through calcineurin. We reasoned that if CsA was acting via calcineurin-dependent signaling, we would lose the enhanced neurosphere formation in cells treated with CsA and cyclophilin A inhibitor. We cultured primary adult NPCs in CsA + inhibitor (range 0–0.25 nM). As predicted, blocking cyclophilin A did not inhibit the pro-survival effects of CsA, further supporting the hypothesis that the pro-survival effects are calcineurin-independent (Fig. 2C).

### NIM811 (*N*-methyl-4-isoleucine CsA) has direct effects on NPCs

**Fig. 3.**
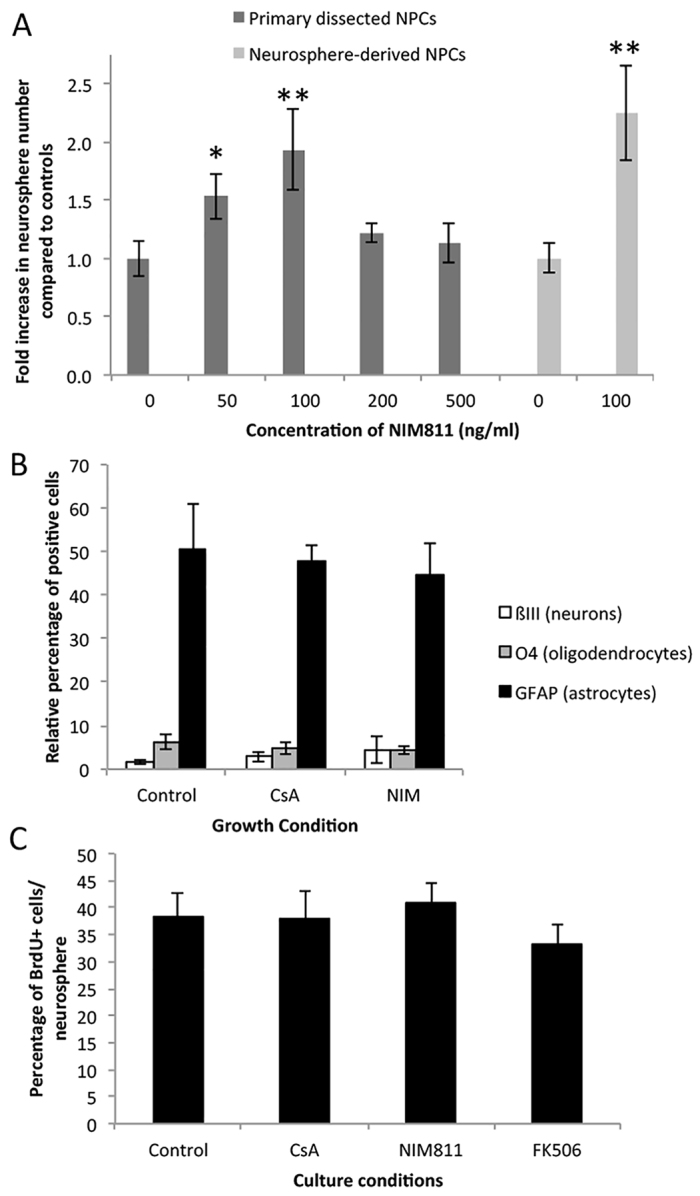
**NIM811 has direct effects on NPCs.** (A) NIM811 was added to primary subependyma-derived cell cultures (*n*=3 independent experiments) or neurosphere-derived cells (*n*=3 independent experiments). In primary cultures (dark gray bars), neurosphere numbers were significantly higher at 50 ng/ml (1.5-fold) and 100 ng/ml (1.9-fold) NIM811. Neurosphere-derived cells cultures (light gray bars) also showed a 1.9-fold increase in neurosphere numbers at 100 ng/ml NIM811. *P<0.05, **P<0.01. (B) Primary neurospheres grown in the presence of no drug, CsA or NIM811 had similar differentiation profiles (*n*=6 neurospheres/group). Individual neurospheres were differentiated and stained for neurons (βIII-tubulin), oligodendrocytes (O4) and astrocytes (GFAP*).* (C) Secondary neurospheres grown in control conditions or in the presence of drug treatment (CsA, NIM811 or FK506) were pulsed with BrdU for 4 hours and revealed no change in proliferation (*n*=12–24 neurospheres per group; *P*>0.05).

NIM811 is a non-immunosuppressive analog of CsA that inhibits MTP pore formation in a calcineurin-independent manner by binding cyclophilin D. Waldmeier et al. demonstrated that NIM811 is equipotent to CsA in blocking MPT pore formation and thereby in reducing apoptotic and necrotic cell death in hepatocytes exposed to pro-apoptotic molecules and damage pathways (Waldmeier et al., 2002). To determine the effects of NIM811 on adult NPCs, we performed the neurosphere assay using a range of NIM811 concentrations (0–500 ng/ml) (Fig. 3A). We observed a significant increase in neurosphere numbers at 50 ng/ml (14±1 neurospheres, 1.5-fold increase; *P*<0.05) and at 100 ng/ml (17±1 neurospheres, 1.9-fold increase; *P*<0.01) relative to control conditions (9±1 neurospheres) (Fig. 3A), with a concomitant increase in neurosphere size (131±9 μm at 50 ng/ml and 124±8 μm at 100 ng/ml NIM811 versus 88±8 μm in control conditions; *P*<0.05). Furthermore, consistent with previous work (Hunt et al., 2010), we demonstrate that neither CsA nor NIM811 alters the differentiation profile of primary neurospheres grown in either drug (Fig. 3B). Most important, we used 100 ng/ml NIM811 on neurosphere-derived cells and found a similar 1.9-fold increase relative to control (22±2 versus 12±1 per 5000 cells, treatment versus control, respectively; *P*<0.05) (Fig. 3A), which demonstrated that NIM811 acts directly on neural stem and progenitor cells. To address the possibility that NIM811 was enhancing proliferation, secondary neurospheres were grown for 4 days in the presence or absence of drug treatment (CsA, NIM811 or FK506) and bromodeoxyuridine (BrdU) was added to the cultures for 4 hours. Individual colonies were collected and plated in individual wells for 2.5 hours on laminin, fixed and stained for BrdU. As shown in Fig. 3C, we observed no significant difference in the relative percentage of BrdU-labeled cells in any of the conditions. The lack of a change in proliferation kinetics seen with NIM811 further supports the conclusion that the pro-survival effects of CsA are mediated through a calcineurin-independent pathway. Hence, through promoting NPC survival by blocking cyclophilin D and MPT pore formation using NIM811, we were able to replicate the pro-survival effects of CsA *in vitro*.

### The pro-survival effects of CsA are independent of nNOS and BAD

**Fig. 4.**
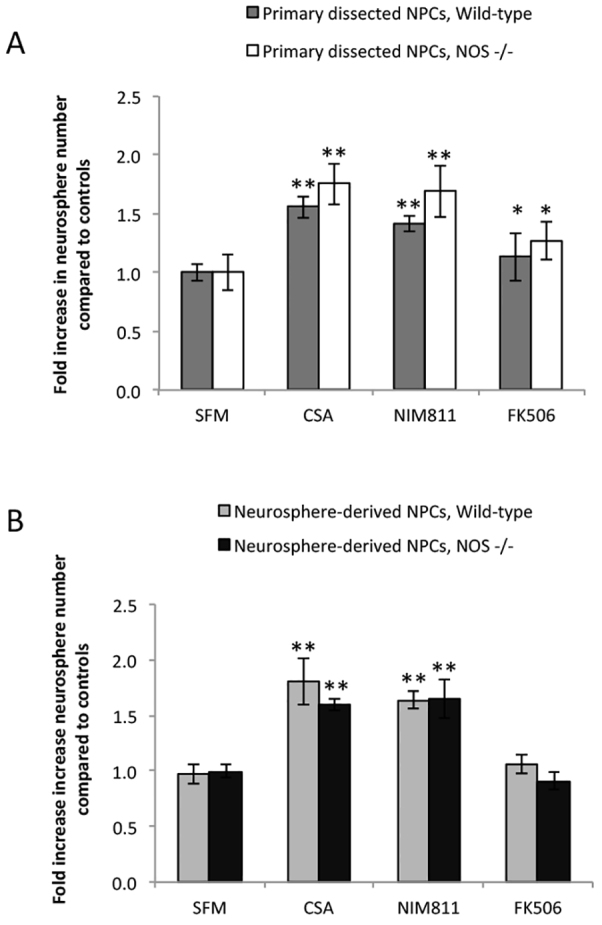
**CsA and NIM811 have similar effects on neurosphere numbers.** (A) CsA, NIM811 or FK506 were added to primary NPC cultures (*n*=3 independent experiments, 2–3 mice/group/experiment). CsA, NIM811 and FK506 exposure resulted in significant increases in the numbers of neurospheres from both *nNOS*^−/−^ and littermate controls relative to untreated control cultures (SFM). (B) CsA, NIM811 or FK506 were added to neurosphere-derived cells (*n*=3 independent experiments, 2–3 mice/group/experiment). CsA and NIM811, but not FK506, significantly increased the numbers of neurospheres from *nNOS*^−/−^ and littermate controls relative to untreated controls (SFM). *P<0.05, **P<0.01.

These findings make the strong prediction that the effects on NPCs mediated by CsA and NIM811 are independent of calcineurin and, as such, independent of the nNOS- and BAD-mediated cell survival pathways downsteam of calcineurin signaling (Fig. 1). To test these predictions, we first performed experiments using mice lacking nNOS activity (*nNOS*^−/−^ mice) (Huang et al., 1993). If nNOS mediated the pro-survival effects of CsA and NIM811, we would observe no change in neurosphere numbers or size derived from *nNOS*^−/−^ mice in the presence of CsA or NIM811 relative to *nNOS*^+/+^ littermate controls. Indeed, we observed significant increases in the numbers of primary and secondary neurospheres in CsA- and NIM811-treated cultures from *nNOS*^−/−^ and *nNOS*^+/+^ (littermates) cultures (Fig. 4A,B), with a concomitant increase in neurosphere size (data not shown). Interestingly, FK506 exposure also revealed a significant increase in primary neurosphere number, suggesting that nNOS does not mediate the pro-survival effects elicited by the non-NPC cells in cultures. Consistent with our findings that FK506 acts indirectly on NPCs, no increase was seen using passaged neurosphere-derived cells. Together, these data support the hypothesis that CsA-mediated pro-survival effects are independent of nNOS signaling.

**Fig. 5.**
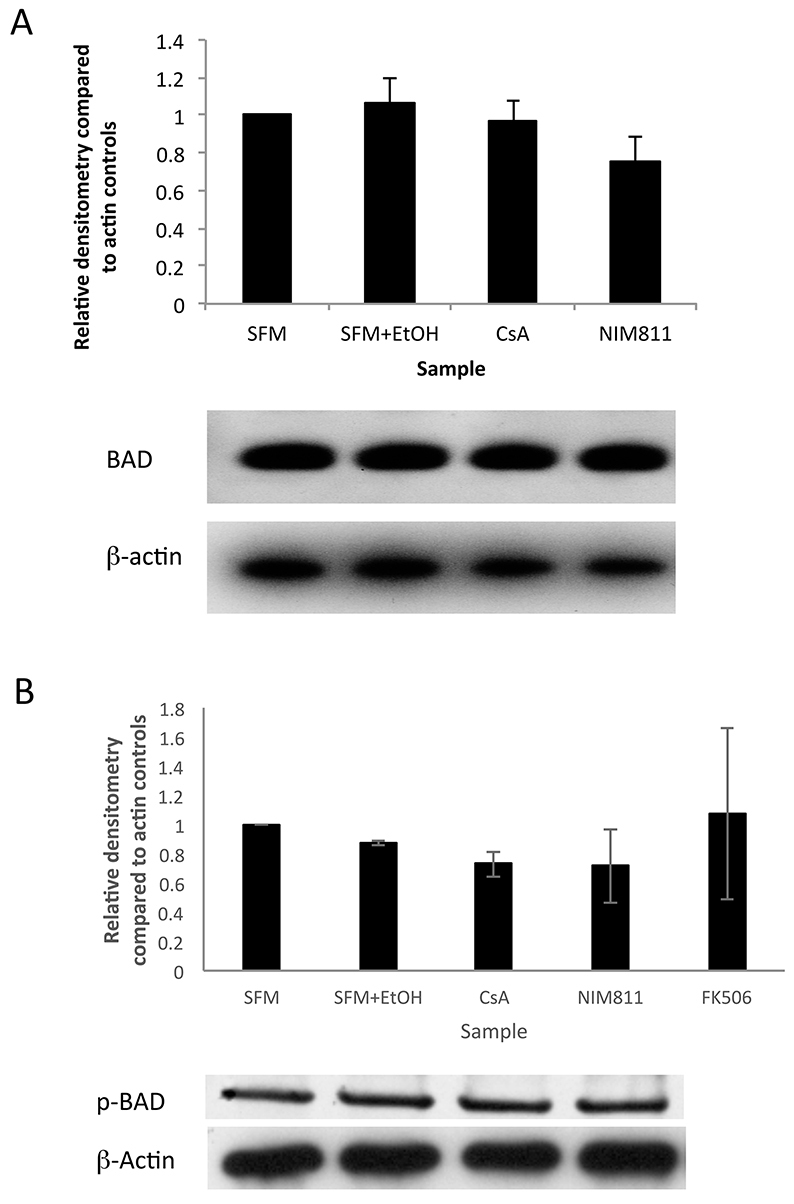
**CsA acts on NPCs via a calcineurin-independent pathway.** (A) Relative expression of phospho-BAD normalized to β-actin controls in primary NPC cultures grown in SFM alone, SFM + solvent control, SFM + CsA (100 ng/ml) or SFM + NIM811 (100 ng/ml) showing no increase in BAD expression with a representative western blot (*n*=4 independent trials, *P*>0.05). (B) Relative expression of BAD normalized to β-actin controls in primary NPC cultures grown in SFM alone, SFM + solvent control, SFM + CsA (100 ng/ml), or SFM + NIM811 (100 ng/ml) showing no increase in phospho-BAD expression (*n*=4 independent trials, *P*>0.05).

Next, we asked whether CsA and NIM811 resulted in phosphorylation of BAD. BAD is dephosphorylated by active calcineurin and this dephosphorylation is inhibited by various signals, including the binding of CsA to cyclophilin A. Therefore, we predicted that if CsA is acting through a calcineurin-dependent pathway, we would observe an increase in phosphorylated BAD and a concomitant decrease in BAD in neurospheres derived from CsA-treated cultures. Primary adult neurospheres were grown in control conditions with or without CsA (100 ng/ml) or NIM811 (100 ng/ml). Western blot analysis demonstrated no increase in the level of phosphorylated BAD or decrease in BAD in CsA or NIM811 cultures (Fig. 5; *P*>0.05) relative to cultures in control medium, consistent with the prediction that CsA and NIM811 are signaling via a calcineurin-independent pathway. Taken together, these results indicate that CsA acts on NPC survival via a calcineurin-independent pathway.

### NPCs respond similarly to CsA and NIM811 *in vivo*, but infusion of FK506 *in vivo* has no effect on NPCs

**Fig. 6.**
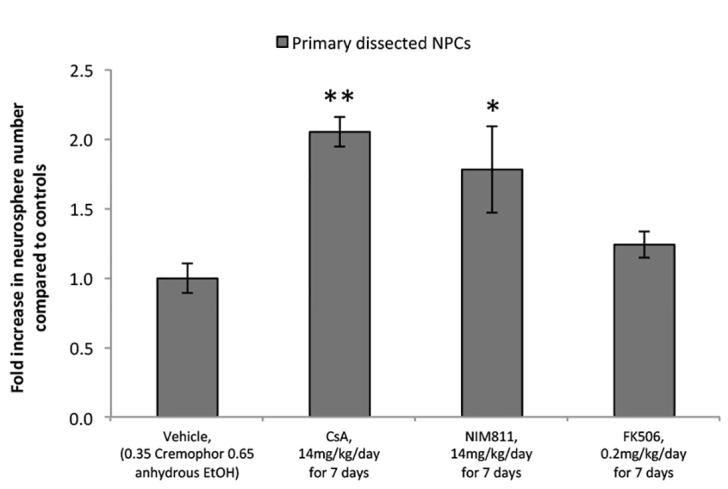
***In vivo* administration of CsA or NIM811, but not FK506, increases the size of the neural stem cell pool.** Increased numbers of neurospheres were seen in primary NPC cultures from mice receiving systemic infusion of CsA or NIM811 for 7 days (*n*=3 independent experiments, 3 animals/group/experiment). *P<0.05; **P<0.01.

We have shown that CsA administration *in vivo* increases the number of the NSCs in the adult brain (Hunt et al., 2010). We asked whether NIM811 would cause similar increases following *in vivo* administration. Adult mice received 7-day infusions of saline, CsA (14 mg/kg/day), FK506 (0.2 mg/kg/day) or NIM811 (14 mg/kg/day) delivered via an osmotic minipump placed subcutaneously. Neurosphere cultures were established and the number of neurospheres was assayed. Consistent with the *in vitro* findings, we observed a significant 2.1-fold and 1.8-fold increase in neurosphere numbers from CsA-infused and NIM811-infused animals, respectively (18.9±1 and 16.4±3 neurospheres versus 9.2±1 neurospheres/5000 cells with CsA, NIM811 and control infused animals, respectively; *P*<0.01 for CsA and *P*<0.05 for NIM811) (Fig. 6). FK506 infusion did not result in an increase in neurosphere formation (11.4±1 neurospheres/5,000 cells) (Fig. 6). Together, these data reveal that the pro-survival of effects of CsA, replicated by NIM811, are mediated by cyclophilin D binding and blocking MPT pore formation.

### NIM811 infusion following ischemic injury results in functional recovery in mice, similar to the effects seen with CsA

We have shown previously that CsA administration following stroke results in functional recovery in mice (Erlandsson et al., 2011). Therefore, we proposed that if NIM811 and CsA act via the same mechanism to inhibit MPT pore formation, we would see a similar functional recovery in mice infused with NIM811 following stroke. To test this, young adult C57/Bl6 mice received an endothelin-1 (ET-1) lesion of the sensory-motor cortex to induce ischemia in the forelimb region and resulting in motor deficits in forelimb function. At the time of stroke, mice received subcutaneous pumps that delivered CsA (14 mg/kg/day), NIM811 (14 mg/kg/day) or vehicle (65% ethanol: 35% cremaphor) until the time of sacrifice at day 32 post-stroke. Forelimb function was assayed using the foot-fault task (Erlandsson et al., 2011). Baseline was established three days prior to stroke and mice were tested on days 4, 11, 18, 25 and 32 post-stroke. Briefly, the number of slips per paw were recorded when mice were placed on an elevated metal wire grid. A deficit was calculated as a positive percentage slippage of the paw contralateral to the ET-1 lesioned hemisphere (normalized to the ipsilateral, uninjured paw and the total number of steps).

**Fig. 7.**
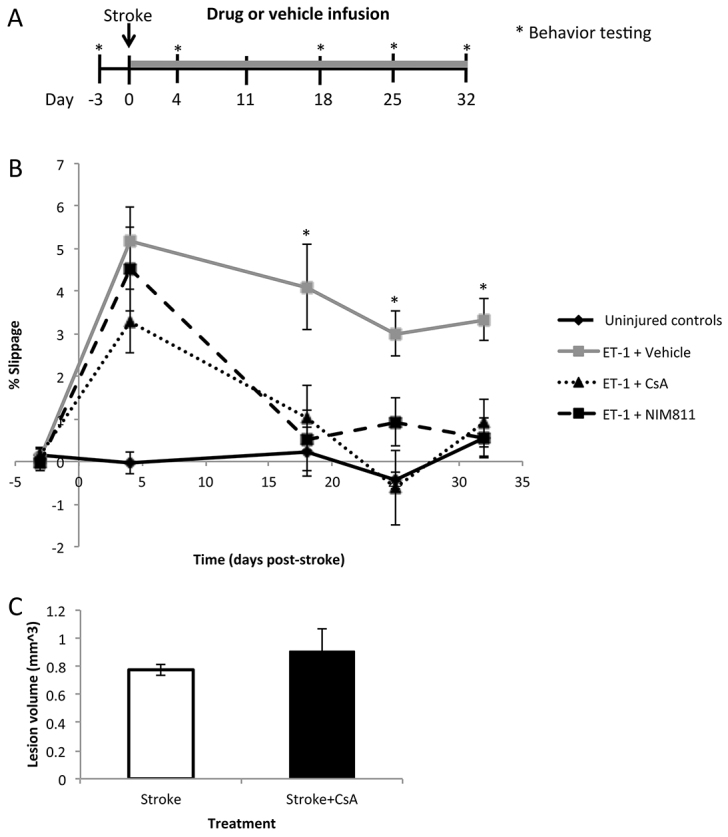
**CsA or NIM811 infusion following stroke results in functional recovery following stroke.** (A) Experimental paradigm for injury, treatment and behavior testing. (B) Animals received ET-1 injections and subcutaneous pumps with CsA (*n*=5 mice), NIM811 (*n*=7 mice) or vehicle (*n*=6 mice). ANOVA and Bonferroni post-hoc analyses reveal that mice receiving NIM811 or CsA were not significantly different from uninjured controls at 18, 25 and 32 days post-stroke, whereas stroke-injured mice receiving vehicle treatment were significantly different from uninjured controls and CsA- and NIM811-infused animals at these same time-points. (C) Lesion volumes were calculated 2 days post-stroke from animals that received stroke alone or stroke+CsA (*n*=3–4 per group); *P<0.05.

Prior to stroke, all mice displayed equal slippage between both forelimbs; however, at 4 days post ET-1 injection, mice displayed a significant functional deficit (*P*<0.05), with increased contralateral slippage relative to uninjured controls. Because CsA and NIM811 administration began at the time of stroke, these significant functional deficits at day 4 suggest that any long-term effects of CsA or NIM811 administration was not the result of neuroprotection in the ischemic cortex. This lack of neuroprotection was supported by an analysis of the developing lesion at 2 days post-stroke, which revealed no difference in the size of the lesion formed between stroke only and stroke+CsA mice (Fig. 7C). As early as day 18 post-stroke, both CsA- and NIM811-treated mice were not significantly different from uninjured controls (*P*>0.05) (Fig. 6), indicating that they had recovered their forelimb use. Additionally, ANOVA demonstrated that both ET-1+CsA and ET-1+NIM811 animals had significantly recovered by day 18 from their stroke deficit at day 4 (*P*<0.05) and not significantly different from their pre-stroke values (*P*>0.05). ET-1 + vehicle animals were still significantly different from uninjured controls (*P*<0.05) at day 18 post-stroke and remained significantly different from controls at all time-points examined. Strikingly, NIM811-infused mice recovered at the same rate and to the same extent as CsA-treated mice (Fig. 7). This parallel recovery provides further support for the hypothesis that the mechanism of action of CsA can be accounted for by inhibition of MPT pore formation targeted by NIM811.

## DISCUSSION

In this study, we have examined the mechanism by which CsA enhances NPC survival using both *in vitro* and *in vivo* models. As seen in Fig. 1, CsA can enhance cell survival in a calcineurin-dependent fashion through binding with cyclophilin A in the cytoplasm or through a calcineurin-independent pathway mediated by CsA binding to cyclophilin D in mitochondria. We have established that NIM811 (which binds with cyclophilin D) but not FK506 (which inhibits calcineurin) has a direct effect on adult NPCs, similar to CsA. Specifically, CsA and NIM811 increase the numbers and size of neurospheres, thus suggesting that NIM811 and CsA share a common pathway. Both NIM811 and CsA increase the size of the NPC pool in mice lacking neuronal nitric oxide synthase, indicating that the NO/NOS pathway, which is downstream of calcineurin, does not mediate the pro-survival effects. The findings that (1) phosphorylated BAD did not change in neurospheres exposed to CsA or NIM811 and (2) blocking cyclophilin A did not inhibit the pro-survival effects of CsA, further confirm that CsA acts through a calcineurin-independent pathway to promote NPC survival. Finally, NIM811 infusion *in vivo* was able to mimic the functional recovery seen with CsA infusion following ischemic stroke. These findings support the conclusion that the increased NPC survival mediated by CsA is calcineurin-independent and due to CsA binding with cyclophilin D, leading to inhibition of MPT pore formation.

FK506 increased neurosphere numbers in primary cultures but not in NPC-derived cultures. We found that conditioned media from FK506-treated primary cultures contained a soluble factor(s) that increased neurosphere numbers in cultures of neurosphere-derived cells. The mechanism by which the exogenous factor acts is independent of neuronal nitric oxide synthase. Although we have previously shown that CsA directly enhances NPC survival (Hunt et al., 2010), we cannot say conclusively whether FK506 increased neurosphere numbers through survival or by changing cell proliferation kinetics. Indeed, Lan et al. derived conditioned media from FK506-treated keratinocytes in cultures and observed enhanced proliferation of both melanocytes and melanoblasts *in vitro* (Lan et al., 2005). Using [^3^H]-thymidine, they demonstrated increased DNA synthesis in melanocytes and melanoblasts and speculated that stem cell factor, which was increased in the FK506-treated keratinocyte cultures, was responsible for the enhanced proliferation. Our observation that conditioned media from FK506-treated primary subependymal cultures led to an increase in the size of the NPC pool is consistent with this finding. Notably however, the concentration of FK506 required to observe an effect on NPCs was significantly higher than the concentration of CsA. The concentration of FK506 would not be used clinically because similar levels of FK506 in the blood would result in overwhelming toxicity (Grimbert et al., 1999). Further investigation into the released factor and specific cell source in primary cultures will be of interest.

The molecules CsA, FK506 and NIM811 have been shown to have neuroprotective effects in various animal models of neurological injury (Kaminska et al., 2004). Our data suggest that CsA does not act in a neuroprotective manner because the lesion site forms in the same way, regardless of the presence or absence of CsA immediately following stroke. NIM811 is a relatively new molecule so its effects, interactions and side effects are not fully established. Interestingly, we found that the *in vivo* administration of NIM811 and CsA but not FK506 (when used at non-toxic concentrations) could increase the numbers of NSCs and their progeny in the uninjured brain. Most strikingly, infusion of CsA and NIM811 into mice that receive an ischemic injury led to functional recovery, similar to previous studies examining endogenous repair strategies in models of stroke (Cooke et al., 2011; Erlandsson et al., 2011; Kolb et al., 2007; Wang et al., 2012). It has been shown that NPC cells are activated following stroke and can migrate to the lesion site, producing mature cell types. Original work using EGF+EPO has shown that new neurons and astrocytes are formed from the migrating NPCs (Kolb et al., 2007). Subsequently, CsA has also been shown to activate NPCs (Hunt et al., 2010) and following stroke they are recruited to the lesion site where they give rise to astrocytes (Erlandsson et al., 2011). In both cases (EGF and EPO infusion and CsA infusion), functional recovery was observed in the stroke-injured animals, suggesting that new neurons are not necessary for the behavioral recovery. The fact that NIM811 can inhibit cell death pathways in NPCs *in vivo* and *in vitro*, and does not have the immunosuppressive effects of CsA, suggests its potential use in regenerative strategies.

The finding that NIM811 can mimic the pro-survival effects of CsA provides insight into the specific molecular mechanism by which CsA promotes functional recovery following stroke and further enables the development of drugs and small molecules to target specific pathways without the negative peripheral side-effects of broader acting drugs.

## MATERIALS AND METHODS

### Animals

All mice were housed in the University of Toronto animal facilities and maintained in accordance with the Institutional guidelines. Adult male CD1 mice (6–8 weeks, 25–30 g; Charles River) and adult male C57/Bl6 mice (8 weeks, Charles River) were delivered one week prior to use. *nNOS*^−/−^ mice (JAX Stock #2986; Jackson Laboratories) were bred in the facility and genotyped for homozygous knockouts using previously described protocols (Jackson Laboratories).

### Tissue preparation and culture

NSCs were isolated by dissection of the forebrain subependyma of adult male CD1 mice as previously described (Morshead et al., 2003). Briefly, tissue was digested with enzymes (1.33 mg/ml trypsin, 0.67 mg/ml hyaluronidase, and 0.2 mg/ml kynurenic acid) (all from Sigma-Aldrich, St Louis, MO) for 40 minutes at 37°C. Enzyme activity was inhibited with 0.67 mg/ml trypsin inhibitor (Roche Diagnostics, Indianapolis, IN) and the tissue was mechanically dissociated into a single cell suspension. Cells were plated at clonal density (5–10 cells/μl) (Coles-Takabe et al., 2008) in 24-well polystyrene plates (VWR, Mississauga, ON) with serum-free medium (SFM) supplemented with epidermal growth factor (20 ng/ml; Sigma-Aldrich), basic fibroblast growth factor (10 ng/ml; Sigma-Aldrich), heparin (7.35 ng/ml; Sigma-Aldrich), and 1% penicillin/streptomycin (Invitrogen, Eugene, OR). Stock solution of CsA (0.2 mg/ml; Bioshop Canada Inc., Burlington, ON) was made by dissolving solid CsA in SFM solution supplemented with 1:1 anhydrous ethyl alcohol:growth factor, and subsequently added to the cultures at various concentrations. NIM811 (C_62_H_111_N_11_O_12_; molecular weight 1202.64 g/mol; Cas number 143205-42-9) was kindly provided by Novartis, Basel, Switzerland. Stock solution of NIM811 (0.2 mg/ml) was made by dissolving solid NIM811 in SFM solution supplemented with 1:1 anhydrous ethyl alcohol:growth factor and added to cultures at various concentrations. FK506 (0.2 mg/ml; Sigma-Aldrich) powder was reconstituted to a stock solution in SFM solution supplemented with 1:1 anhydrous ethyl alcohol:growth factor and added to cultures at various concentrations. Stocks were made fresh from solid forms of small molecules and not stored for any length of time. Cyclophilin A inhibitor (CAS 1186372-20-2; EMD Millipore) was reconstituted to a stock solution of 10 μM in DMSO and stored in small aliquots at −20°C until use. Stock aliquots were subsequently diluted to final concentrations for dose-response curves in supplemented SFM or SFM+CsA. For experiments using neurosphere-derived NPCs, bulk cell cultures were passaged by pelleting the sample and mechanically dissociating the pellet using a small borehole pipette and growth factor-supplemented SFM and plated as described.

### Conditioned media

NPC cultures were established from primary dissected lateral ventricle tissue as described above. Cells were plated at high density (30 cells/μl). Three conditioned media were prepared: cells plus 2000 ng/ml FK506; cells only; and 2000 ng/ml FK506 only. After 24 hours incubation at 37°C, medium from each culture was collected separately according to the following protocol. Conditioned medium was centrifuged at 100 ***g*** for 5 minutes. The supernatant was aspirated, processed using a 0.22-μm cell strainer and collected in a clean tube. The media were supplemented with EGF, FGF and heparin at concentrations described above. Neurosphere-derived cells were plated at a density of 5 cells/μl in growth factor-supplemented control or conditioned media. The neurospheres that formed were counted 7 days later.

### Neurosphere differentiation

For differentiation, individual neurospheres were plated onto laminin-coated 48-well plates in SFM with 1% fetal bovine serum. After 7 days *in vitro*, cells were fixed with 4% PFA for 20 minutes at room temperature and then washed with PBS.

### BrdU pulse

Primary neurospheres grown in SFM alone were passaged and re-plated at 10 cells/μl in the presence of CsA, NIM811, FK506 or control conditions as previously described. Secondary neurospheres were allowed to form for 4 days and then 10 μM BrdU (Sigma) was added to neurospheres for 4 hours at 37°C. Subsequently, individual neurospheres were picked and plated onto Matrigel-coated 48-well plates and allowed to settle for 2.5 hours at 37°C. Plates were spun at 1500 rpm for 10 minutes and then fixed with 4% PFA for 20 minutes at room temperature, then washed with PBS. Whole neurosphere images were taken at 20× magnification on an AxioVision Zeiss UV microscope. Total number of BrdU-positive cells were counted and compared with the total number of cells within the neurosphere.

### Immunohistochemistry

For immunohistochemistry, fixed neurospheres were permeabilized with 0.3% Triton-X in PBS for 20 minutes at room temperature. For BrdU imaging, DNA was denatured with 1 N HCl at 65°C for 30 minutes and then washed in PBS. Neurospheres were blocked with 10% NGS (normal goat serum) in PBS for 1 hour at room temperature. Primary antibodies were incubated at 4°C overnight. The primary antibodies were mouse anti-BrdU (DAKO, MO744, 1:200), mouse anti-GFAP (Sigma, G9629, 1:500), mouse anti-O4 (R&D Systems, MAB1325, 1:1000), and rabbit anti-βIII tubulin (Covance, PRB-435P-100, 1:1000). Secondary antibodies were Alexa Fluor antibodies (Invitrogen): 488 goat anti-rabbit IgG (1:400), 568 goat anti-mouse IgM (1:400) and 647 goat anti-mouse IgG (1:400). Nuclei were counterstained with Hoechst 33258 (Sigma). Five fields of view were imaged per neurosphere for differentiation studies at 20× magnification on an AxioVision Zeiss UV microscope.

### Western blot

Primary NPC cultures were isolated as described above and plated in tissue-culture-treated flasks (BD Falcon) at clonal density (10 cells/μl). After 7 days in culture, neurospheres were collected, protein isolated using RIPA buffer (Cell Signaling Technologies) and frozen at −80°C until use. Protein was run on a SDS-PAGE gel for 2 hours at 100 V and subsequently transferred to a PVDF membrane for 2 hours at 10 V, which was then blocked using 5% milk powder in TBST for 1 hour and incubated overnight with primary antibody S112 phospho-BAD (1:8000, Cell Signaling Technology), ab32445 BAD (1:5000, Abcam) or β-actin (1:25000, Sigma-Aldrich) at 4°C. Horseradish peroxidase substrate solution (Thermo Scientific) was added and the membrane was subsequently imaged on film. Film was scanned and analyzed using ImageJ (Rasband, W.S., US National Institutes of Health). For each sample, the density was first normalized to a control sample and then to that of the loading control (β-actin). Values are expressed as averages ± s.e.m.

### *In vivo* studies

Adult CD1 mice were used for small molecule administration. Mice were administered one of four treatments: (1) CsA (14 mg/kg/day); (2) FK506 (0.2 mg/kg/day); (3) NIM811 (14 mg/kg/day); and (4) vehicle. The drugs were delivered subcutaneously via an osmotic minipump (Alzet Osmotic Pumps, Cupertino, CA) (*n*=4 mice/CsA or saline/s.c). The pump delivered drug at a rate of 0.5 μl/hour for 7 days. NSCs were isolated by dissection of the forebrain subependyma as described above. The cells were plated at a density of 5 cells/μl in growth factor-supplemented SFM. The neurospheres that formed were counted on day 7 of culture.

### Stroke and drug administration

C57/Bl6 mice were anesthetized using isofluorane and injected with ketoprofen (3 mg/kg). Bregma was visualized and a small hole was drilled through the skull at 2.25 mm lateral to the midline and 0.6 mm anterior to Bregma. A 26-gauge Hamilton syringe with a 45 degree beveled tip was inserted 1 mm ventral from the surface of the brain and 1 μl of 400 pmol of Endothelin-1 (ET-1, Calbiochem) was injected at a rate of 0.1 μl/minute. The needle was left in place for 10 minutes after ET-1 injection to prevent backflow and then slowly withdrawn. Mice receiving CsA or NIM811 were implanted at the time of stroke with a subcutaneous mini-osmotic pump (Alzet; 1002; 0.25 μl/hour for 2 weeks) overlying the shoulder blade and lateral to spine. Pumps were changed every 2 weeks until sacrifice. All mice recovered under a heat lamp with a subcutaneous injection of warmed saline.

### Lesion volume analysis

Mice were sacrificed with an overdose of sodium pentobarbital and perfused transcardially with ice-cold PBS followed by 4% PFA. Brains were post-fixed overnight at 4°C then cryoprotected in 20% sucrose until sectioning. Coronal sections (30 μm) were cut on a cryostat (−20°C) through the entire lesion site. Every 5th section through the entire lesion site was imaged. Each section was measured using ImageJ. The volume was calculated using half of an ellipsoid formula where *V*=4/3π *r*^1^*r*^2^*r*^3^ using ½ width = *r*^1^, depth = *r*^2^, ½ (number of sections with lesion sites in a set × 30 μm) = *r*^3^.

### Behavior testing

Mice were given 1 week to acclimate to the animal facility upon arrival. Subsequently, behavior testing occurred weekly, beginning 3 days prior to stroke until sacrifice. All testing was performed in the same biosafety cabinet and at the same time of day (mid-afternoon). Mice were housed identically with clear, red dome houses, one nestlet and food pellets and water *ad libitum*. Testing and scoring was performed blinded to treatment. For the foot fault task, an elevated grid of 1 cm × 1 cm squares was filmed from below. Mice were allowed to explore the grid for 3 minutes and then returned to the home cage. Films were scored for total steps taken, and left paw and right paw slips were counted during slow-motion replay of the tape. Foot faults were assayed by percentage slippage calculated by (contralateral slips – ipsilateral slips/total steps)×100.

### Statistical methods

Statistical analysis was performed using the Student’s *t*-test for two-group comparisons and ANOVA for multiple group comparisons with Bonferroni post-hoc test (SSPS, SigmaStat statistical package or Graphpad Prism). All data are reported as means ± s.e.m.
